# Investigation of La_1−*x*_Sr*_x_*CrO_3−*∂*_ (*x* ~ 0.1) as Membrane for Hydrogen Production

**DOI:** 10.3390/membranes2030665

**Published:** 2012-09-11

**Authors:** Yngve Larring, Camilla Vigen, Florian Ahouanto, Marie-Laure Fontaine, Thijs Peters, Jens B. Smith, Truls Norby, Rune Bredesen

**Affiliations:** 1 SINTEF Materials and Chemistry, P.O. Box 124 Blindern, Oslo NO-0314, Norway; Email: yngve.larring@sintef.no (Y.L.); florian.ahouanto@sintef.no (F.A.); marie-laure.fontaine@sintef.no (M.L.F.); thijs.peters@sintef.no (T.P.); 2 Centre for Materials Science and Nanotechnology, Department of Chemistry, University of Oslo, FERMiO, Gaustadalleen 21, Oslo NO-0349, Norway; Email: camilla.vigen@smn.uio.no (C.V.); truls.norby@kjemi.uio.no (T.N.); 3 Statoil, Forskningsparken, Porsgrunn NO-3908, Norway; Email:jbsm@statoil.com

**Keywords:** hydrogen transport membrane, proton permeation, oxygen permeation, water splitting

## Abstract

Various inorganic membranes have demonstrated good capability to separate hydrogen from other gases at elevated temperatures. Hydrogen-permeable, dense, mixed proton-electron conducting ceramic oxides offer superior selectivity and thermal stability, but chemically robust candidates with higher ambipolar protonic and electronic conductivity are needed. In this work, we present for the first time the results of various investigations of La_1−*x*_Sr*_x_*CrO_3−*∂*_ membranes for hydrogen production. We aim in particular to elucidate the material’s complex transport properties, involving co-ionic transport of oxide ions and protons, in addition to electron holes. This opens some new possibilities for efficient heat and mass transfer management in the production of hydrogen. Conductivity measurements as a function of *p*H_2_ at constant *p*O_2_ exhibit changes that reveal a significant hydration and presence of protons. The flux and production of hydrogen have been measured under different chemical gradients. In particular, the effect of water vapor in the feed and permeate gas stream sides was investigated with the aim of quantifying the ratio of hydrogen production by hydrogen flux from feed to permeate and oxygen flux the opposite way (“water splitting”). Deuterium labeling was used to unambiguously prove flux of hydrogen species.

## 1. Introduction

**Figure 1:**
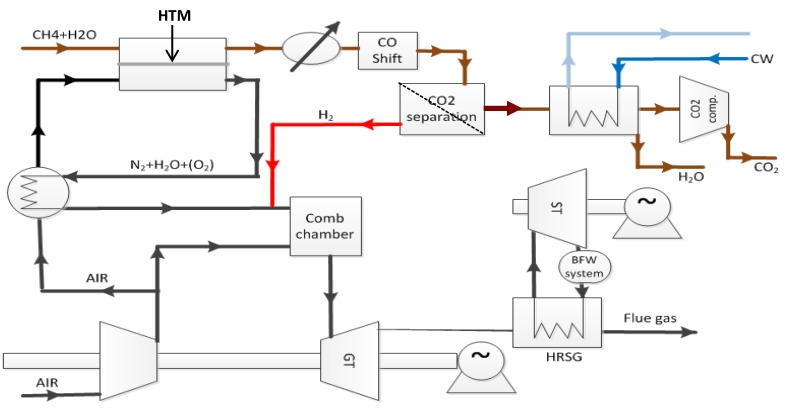
PCDC process suggested by Statoil with integrated ceramic mixed conductor membrane [3].

Current concern about climate change has generated large efforts to reduce CO_2_ emissions. Hydrogen selective membranes are envisaged as a key enabling technology in various processes to lower energy consumption and for capturing CO_2_. Notably, inorganic hydrogen separation membranes have been studied for a long time for applications in so-called pre-combustion decarburization (PCDC) processes in power generation with CO_2_ capture. Since the various types of hydrogen separation membranes cover an operation window from ambient to ~1000 °C, tailor-making of process schemes, conditions and membrane properties are both a challenge and a great possibility for improving energy efficiency and creating sustainable operation. One may currently find various conceptual integration possibilities aiming to bridge the gap between process operation window and membrane properties [1,2]. An important aspect of this integration is to ensure sufficient driving force for the flux. For dense metallic or porous hydrogen separation membranes, the driving force for flux is proportional to 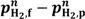 where f and p refer respectively to the feed and permeate side, and *n* is typically between 0.5 and 1 for dense metal membranes and 1 for porous membranes. For these, the driving force is the magnitude in the difference between the two pressures, dominated by the largest one, at the feed side. For dense ceramic membranes, however, the flux can be various functions of the hydrogen partial pressures over the membrane, commonly proportional to 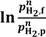, and in this case, it is beneficial to generate the highest possible ratio of the partial pressures of hydrogen over the membrane. Surface limiting effects may change these relationships and decrease the flux. In the concept of Norsk Hydro (now merged with Statoil), see Figure 1, a ceramic mixed conductor membrane for hydrogen separation is integrated at high temperature (900–1000 °C) in the reforming reaction, where a high driving force is sustained by keeping the permeate side at a very low partial pressure of hydrogen by reaction with oxygen in air [3]. The purpose of the membrane is three-fold: (i) The membrane separates the two gas streams of natural gas (feed side) and air (permeate side). Hydrogen is transported from the feed side to the permeate side where it reacts with oxygen to generate heat to sustain the endothermic reforming process. The oxidation of hydrogen keeps the hydrogen partial pressure very low at the permeate side, which, as mentioned above, is particularly beneficial for the driving force for flux. (ii) Only the required amount of air required for heat generation is used, thus the permeate stream leaving the reactor is rich in N_2_. Hence, the membrane process enables N_2_ co-production that is required to dilute the hydrogen fuel for the subsequent gas turbine combustion process. (iii) Finally, the thin membrane acts as a heat exchanger material.

In dense ceramic hydrogen transport membranes (HTMs), one utilizes the mixed conductivity by electrons and protons to make the material permeable to hydrogen gas. The main difficulty for industrial deployment of HTMs lies in the identification of materials combining high proton concentrations and mobility at high temperature, high electron conductivity, and stability towards CO_2_ [4,5]. To tackle these criteria, one may look for materials with mixed valence and modest band gaps in order to have electronic defects. But, first and foremost, one must look at proton concentration in terms of hydration thermodynamics. Nowadays, computational chemistry can predict hydration enthalpies quite reliably [6]. Moreover, there are some empirical correlations for classes of oxides. Hence, perovskites are shown to exhibit more favorable hydration thermodynamics the lower their structural tolerance factors and the more similar the electronegativities are of the A and B site cations [7,8,9,10]. In other words: the more stable the perovskite structure, the fewer the protons at high temperature; the material prefers oxygen vacancies as positive charge carriers charge compensating acceptor dopants, and exhibits essentially oxide ion transport. 

The prime candidates for HTMs early on were based on SrCeO_3_ [11] and related perovskites. Their composites with metals, such as Pt (for higher electronic transport) have also been investigated. As SrCeO_3_ and related perovskites show poor thermodynamic stability and high reactivity with CO_2_, there has been a long search for new and more stable materials, preferably without Sr or Ba as main components, in order to have sufficient stability towards acidic gases like CO_2_ [5]. At present, a few new materials have emerged as promising candidates, such as La_6_WO_12_, with stable compositions in the range La_6−*x*_WO_12−3*x*/2_ (6−*x* = 5.3−5.7) [12].

In the search of potential materials for their PCDC process, Statoil patented a range of alternative, stable perovskites as HTMs [13] and suggested how they could be used in natural gas-fired power plants with carbon capture [14]. One of the materials was based on LaCrO_3_. The choice may be surprising because this perovskite should contain few protons according to the aforementioned correlations, which predict LaCrO_3_ to have an endothermic hydration enthalpy of 12 kJ/mol. The argument in favor of this oxide was that the three d-electrons in Cr^3+^ make this ion particularly stable in an octahedral crystal field, favoring a filled oxygen lattice with protons (as OH^−^ ions) over oxygen vacancies to a larger extent than expected from the general trend. Statoil optimized the composition for sinterability, developed porous tubular supports with dense thin layers of the material, and tested their use for hydrogen extraction. The hydration and hydrogen transport were investigated in collaborative projects with the University of Oslo and SINTEF over a number of years. During the work, it has become clear that the transport properties utilized in the PCDC process are more complex than anticipated. The ionic transport in the material appears to have contributions both from oxide ions and protons. This feature adds an interesting side effect of the membrane related to the operation condition in Figure 1; namely that the membrane also contributes to partial oxidation and steam generation at the feed side by transporting oxygen from the permeate side, creating heat on the reforming side as well. The significance of this effect has not yet been fully explored, and should be a subject of a separate study; but it is interesting to note that the effect could reduce the need for the addition of steam to the feed stream since it is internally generated. 

In this study, the transport properties, including hydrogen species of ceramic membranes of Sr-substituted LaCrO_3_ (LSC), are reported for the first time. We summarize the work done with LSC membranes—including hydrogen permeation as well as concentration and mobility of protons—based on formerly undisclosed results and new measurements. We shall see that LSC does indeed dissolve modest amounts of protons and exhibits proton conductivity mixed with p-type electronic and oxide ion conductivities, all depending on temperature, *p*O_2_, and *p*H_2_O. Production of hydrogen using such a material as a membrane towards a hydrogen-rich mixture stems partly from mixed protonic-electronic conduction, but may have a contribution from splitting of any water vapor in the permeate sweep gas, based on mixed oxide ion-electron conduction, and the two phenomena may be difficult to distinguish. 

## 2. Brief Review of Undoped and Acceptor-Substituted LaCrO_3_

### 2.1. Properties and Applications

Lanthanum chromite, LaCrO_3_, is a refractory oxide with melting point of 2430 °C, taking on a perovskite-related structure with orthorhombic symmetry (*Pnma*, space group 62) [15]. It exhibits a small range of cation non-stoichiometry, as indicated in the phase diagram [16], and it can be substituted with a number of homo- and heterovalent cations on both the A (La) and B (Cr) sites. Of greatest interest is the effect of acceptor substitution by alkaline earth cations—notably Ca^2+^ or Sr^2+^—for La^3+^ [17,18], causing charge compensation by oxygen vacancies at low *p*O_2_ and electron holes at high *p*O_2_ [19,20,21]. For this reason, Ca- and Sr-substituted LaCrO_3_—hereafter LCC and LSC, respectively—exhibit high p-type electronic conductivity under oxidizing and moderately reducing conditions, falling off under more reducing conditions, but still with dominating p-type electronic conductivity well above 1 S/cm [19,22,23]. This, along with its great stability towards oxidation as well as reduction, has made it of interest as an interconnect material in SOFCs, where it must exhibit electronic conductivity, be gas tight in order to separate the fuel and oxidant, and preferably not conduct oxide ions [19,24].

It was pointed out early on that the presence of oxygen vacancies would give considerable oxide ion conductivity and permeability of oxygen in LCC and LSC [22], but it appeared that the thickness of the interconnect and the rather slow surface kinetics of LaCrO_3_-based materials made this a minor problem [30].

It is very difficult to sinter LaCrO_3_-based ceramics to high density, and a lot of research has been invested into overcoming this bottleneck, using, for instance, sintering aids [25]. Interconnects of these materials also suffer from Cr evaporation, notably by hexavalent chromium oxohydroxides [26], which poisons SOFC cathodes by the deposition of Cr oxides at the electrocatalytic triple-phase boundary sites between strontium-substituted LaMnO_3_ (LSM) cathodes and YSZ electrolytes [27]. Additional weaknesses comprise moderate mechanical properties, chemical expansion by reduction, limited thermal conductivity, and high costs of machining. For these reasons, LaCrO_3_-based interconnects are now for the most part being replaced by metallic interconnects—enabled by the reduced temperatures of operation of modern SOFCs [27,28,29]. 

### 2.2. Defect Structure

LaCrO_3_ compensates acceptor-dopants with electron holes under oxidizing conditions and oxygen vacancies under reducing conditions, accompanied by corresponding increases the oxygen ion and p-type electronic conductivities. Studies, where oxygen non-stoichiometry has been measured as a function of oxygen partial pressure (*p*O_2_) by means of thermogravimetry, have been conducted for LaCrO_3_ with various dopants, doping levels and temperatures [20,21]. Mizusaki *et al.* [20] reported that the oxygen non-stoichiometry *δ* for La_1−*x*_Sr*_x_*CrO_3−*δ*_ (*x* = 0.1–0.3) was close to zero for *p*O_2_ > 10^−5^ and eventually reached *x*/2 with decreasing *p*O_2_. These *p*O_2_ regimes represent two limiting conditions where the acceptor is predominately charge-compensated by electron holes (*i.e.*, 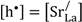) and oxygen vacancies (*i.e.*, 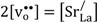), respectively. Moreover, the limiting *p*O_2_, at which oxygen vacancies and electron holes are equally dominant, shifts to higher *p*O_2_ with increasing doping levels and temperatures. The formation of oxygen vacancies at the cost of electron holes can, in Kröger-Vink notation, be written:

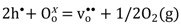
(1)

The equilibrium constant for this reaction is given by:

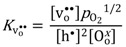
(2)
and Mizusaki *et al.* estimated the standard formation enthalpy of oxygen vacancies according to this reaction to 303 kJ/mol for *x* = 0.1. A similar behavior was observed for LaCr_(__1−*x*)_Mg*_x_*O_3−*δ*_ [21], where the corresponding enthalpy was seemingly independent of dopant concentration and estimated at 272 kJ/mol for *x* = 0.02–0.1.

**Figure 2:**
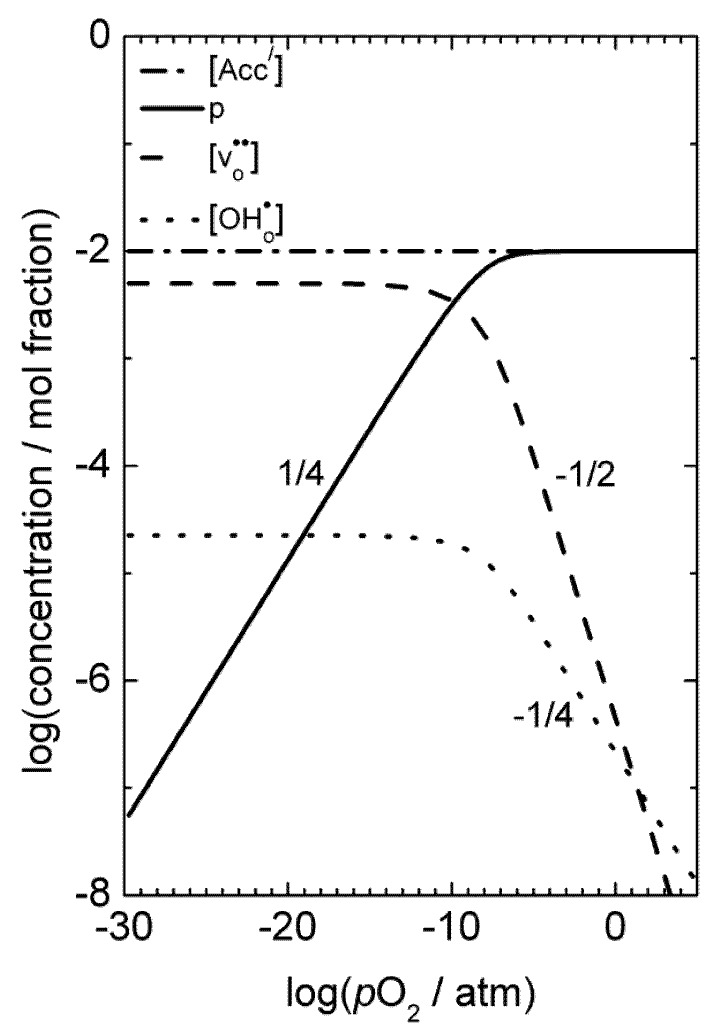
Example of defect concentrations for LSC with 1 mol% acceptor substitution as a function of *p*O_2_ at constant *p*H_2_O.

From the limiting electroneutrality condition, the concentration of electron holes will be independent of *p*O_2_ when the acceptor is charge-compensated by electron holes, since it follows from Equation (2) that it exhibits a *p*O_2_^1/4^-dependecy when the acceptor is charge-compensated by oxygen vacancies. The defect concentrations as a function of *p*O_2_ are illustrated in Figure 2. Due to higher mobility of electron holes in general compared to ionic defects, the total conductivity will also exhibit the *p*O_2_-dependencies of the electron holes [22,23].

### 2.3. A Theoretical Evaluation of Protons in LSC

Hydration of oxygen vacancies in oxides occurs according to:

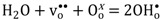
(3)
with the corresponding equilibrium constant:

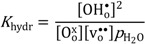
(4)

When protons are included, the overall electroneutrality condition of LSC can be approximated by:

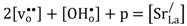
(5)

As can be deduced from Equations (1) and (3) and seen in Figure 2, the concentration of protons increases with decreasing *p*O_2_. From Equation (4), it follows that the concentration of protonic defects as a function of water vapor partial pressure (*p*H_2_O) is proportional to *p*H_2_O^1/2^ if electron holes or oxygen vacancies are the dominating charge compensating defects (and thus independent of *p*H_2_O). If protons become the dominating charge compensator, the minor concentration of oxygen vacancies becomes proportional to *p*H_2_O^−1^, while the concentration of electron holes, via Equation 2, becomes proportional to *p*H_2_O^−1/2^. 

**Figure 3:**
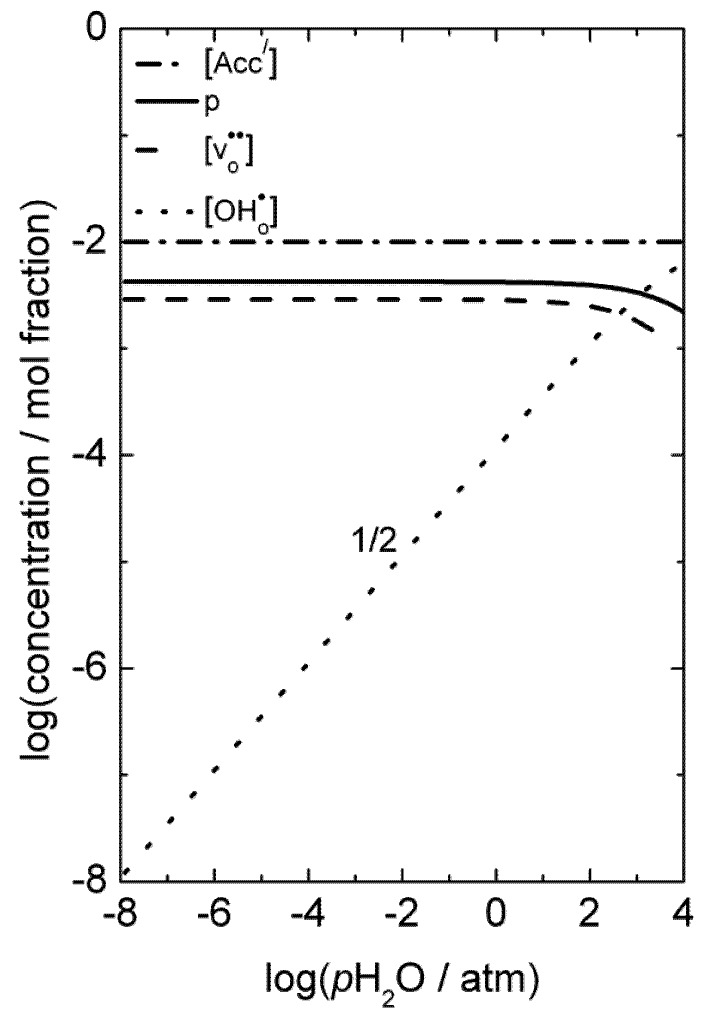
Example of defect concentrations for LSC with 1 mol% acceptor substitution as a function of *p*H_2_O at constant *p*O_2_.

To the best of our knowledge, there have been no significant reports of protons in LSC until this study, and, as mentioned above, the correlation between the enthalpy of the hydration of oxygen vacancies and the stability of the perovskite does not predict that hydration is favorable at all, while the three d-electrons of Cr^3+^ suggests that LSC may be an exception. The defect concentrations as a function of water vapor partial pressure are illustrated in Figure 3, based on a moderately favorable hydration enthalpy. One may note that at high *p*H_2_O, hydration and the presence of protons should be detectable as a decrease in the p-type electronic conductivity as a function of *p*H_2_O.

### 2.4. Transport Properties

The electron holes in LaCrO_3_ appear to behave as small polarons localized to the Cr-site, and the hole mobility exhibits a small activation energy. Consequently, the p-type conductivity is also activated (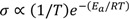), and in the region where the holes are dominating and their concentration given by the constant concentration of acceptors, *E*_a_, is given only by that of the mobility. Karim and Aldred [31] reported that the electrical conductivity of La_1-*x*_Sr*_x_*CrO_3_ increased from the order of 10^−2^ S/cm at temperatures above 1000 °C and *p*O_2_ = 1 atm for *x* = 0, to values two orders of magnitude higher for high values of x up to the maximum level (*x* = 0.4). The electron hole activation energy was estimated at 0.192 eV for the undoped chromite (*x* = 0), and decreased with increasing acceptor-concentrations to 0.113 eV for *x* = 0.4.

LaCrO_3_ is also an oxide ion conductor when acceptor doped. Oxygen vacancy diffusion coefficients in the order of 10^−6^–10^−5^ cm^2^/s were calculated from electrical measurements of LaCrO_3_ with varying dopants and doping levels (0.1–0.35 mol%) at temperatures 900–1100 °C [22]. Based on electrochemical methods, Yokokawa *et al.* [32] estimated a lower vacancy diffusion coefficient in the order of 10^−7^ cm^2^/s at 1000 °C, in agreement with calculations by Van Hassel *et al.,* based on oxygen permeation [33]. Later on, Kawada *et al.* [34] revisited this method and estimated an oxygen vacancy diffusion coefficient in the order of 10^−5^ cm^2^/s. The lower values of the oxygen vacancy diffusion coefficients reported earlier were mainly explained by an underestimation of the oxygen non-stoichiometry in the high *p*O_2_ region.

## 3. Results and Discussion

### 3.1. Conductivity *vs.* pH_2_O

**Figure 4:**
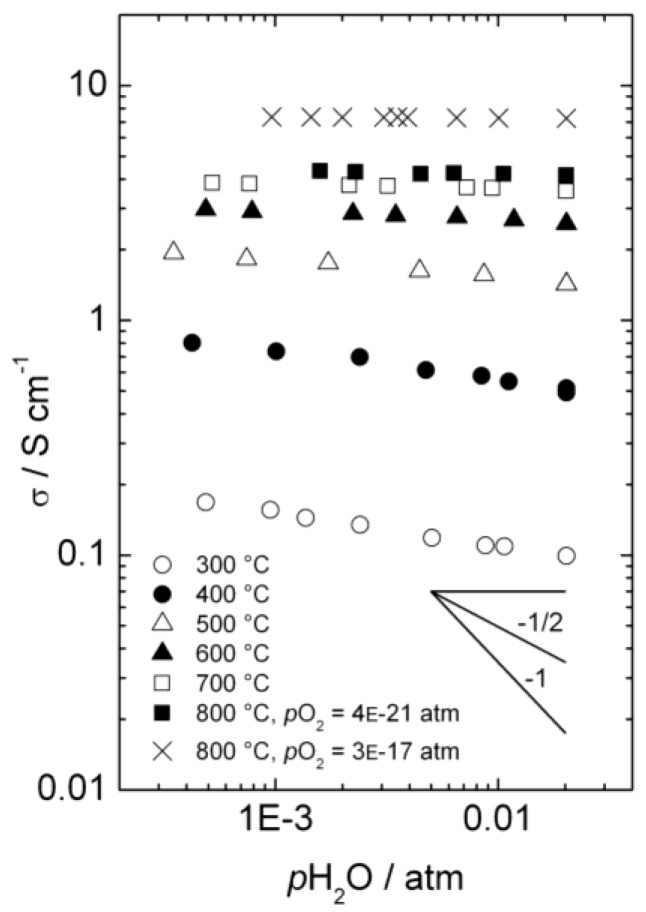
Total a.c. conductivity as a function of water vapor partial pressure for La_0.9_Sr_0.1_CrO_3−*δ*_ in reducing atmospheres.

Figure 4 presents the total a.c. conductivity of La_0.9_Sr_0.1_CrO_3−*δ*_ as a function of *p*H_2_O in reducing atmospheres at temperatures from 300 to 800 °C. Each isotherm is made by diluting a mixture with fixed *p*H_2_/*p*H_2_O ratio with dry Ar, thus keeping *p*O_2_ essentially constant, while changing *p*H_2_O (and *p*H_2_). The values of *p*O_2_ vary from 3 × 10^−17^ and 4 × 10^−21^ atm (depending of *p*H_2_/*p*H_2_O ratio) at 800 °C to 2 × 10^−42^ atm at 300 °C, and under these conditions we may assume that the acceptor dopant is, to a first approximation, charge-compensated by oxygen vacancies. This assumption is in agreement with the higher p-type electronic conductivity observed at *p*O_2_ = 3 × 10^−17^ than at *p*O_2_ = 4 × 10^−21^ atm at 800 °C, as the concentration of electron holes will increase with increasing *p*O_2_ when in the minority. Still, electron holes dominate the total conductivity, due to higher mobility than for ionic defects.

Below approximately 600 °C, the conductivity decreases with increasing *p*H_2_O, reflecting a decrease in the concentration of electron holes as protonic defects are introduced in significant numbers compared to the dominating oxygen vacancies.

**Figure 5:**
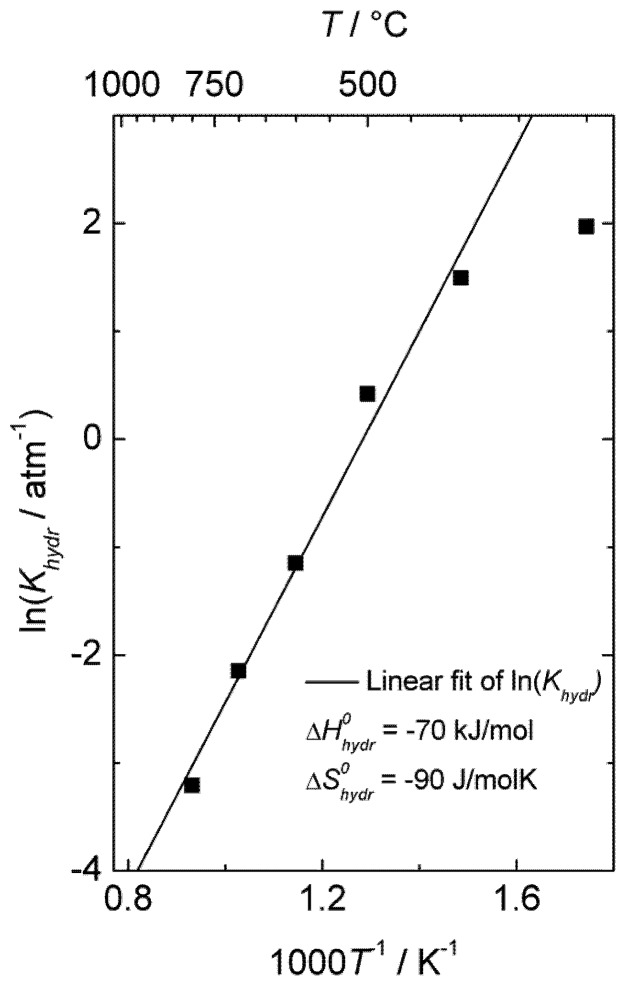
Van 't Hoff plot of hydration equilibrium constant *K_hydr_* for La_0.9_Sr_0.1_CrO_3−*δ*_ corresponding to conductivity data of Figure 4.

By assuming that oxygen vacancies and protons are the dominating charge compensators (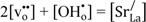), the equilibrium constant of Equation (3), *K_hydr_*, was extracted from the fit of the conductivity curves for each temperature. The results are presented in a van 't Hoff plot (Figure 5), and linear regression of ln*K_hydr_* as a function of 1/*T* yields standard enthalpy and entropy changes of the hydration reaction of −70 kJ/mol and −90 KJ/mol, respectively. The equilibrium constant extracted from conductivity measurements at 300 °C was excluded in this fitting, due to significant deviation from the values obtained at higher temperatures, likely a consequence of slow kinetics. The exothermic hydration enthalpy of −70 kJ/mol for La_0.9_Sr_0.1_CrO_3−*δ*_ is far from the endothermic expected, based on the already mentioned empirical correlation [7,8,9,10], and the aforementioned crystal field stabilization of the d-electrons in fully *vs*. only partially occupied octahedra of the perovskites is an interesting input to understanding this and the many other variations from the correlation. 

### 3.2. Estimated Defect Concentrations and Partial Conductivities

With the parameters obtained by curve-fitting of the hydration equilibrium constant, we have estimated the concentrations of the defects in La_0.9_Sr_0.1_CrO_3−*δ*_ in wet reducing atmospheres (*i.e.*, *p*H_2_O = 0.025 atm, *p*H_2_ = 1 atm). All charge carriers were assumed to follow an activated transport process, *i.e.*, 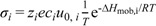. For oxygen vacancies, the pre-exponential factor of mobility, *u*_0*,vo*_, and the enthalpy of mobility, Δ*H_mob,vo_*, were set to 200 cm^2^ K/Vs and 90 kJ/mol, respectively, in accordance with oxygen vacancy diffusion coefficients obtained at temperatures from 950–1100 °C for La_0.8_Sr_0.2_Cr_0.95_Ni_0.05_O_3−*δ*_ [30]. For protons, the pre-exponential factor of mobility, *u_0,OH_*, was set to 13 cm^2^K/Vs, in accordance with values obtained for similar perovskite oxides from [9] and the data set of [8], and the enthalpy of mobility, Δ*H_mob,OH_*, was set to 60 kJ/mol, from the empirical 2/3 of the value for oxygen vacancies [8]. The pre-exponential factor of hole mobility, *u*_0*,h*_, and the enthalpy of hole mobility, Δ*H_mob,h_*, were set in accordance with Weber *et al.* [35] to 200 cm^2^K/Vs and 12.5 kJ/mol, respectively. Furthermore, it was assumed that the nominal Sr acceptor level was effective. 

**Figure 6:**
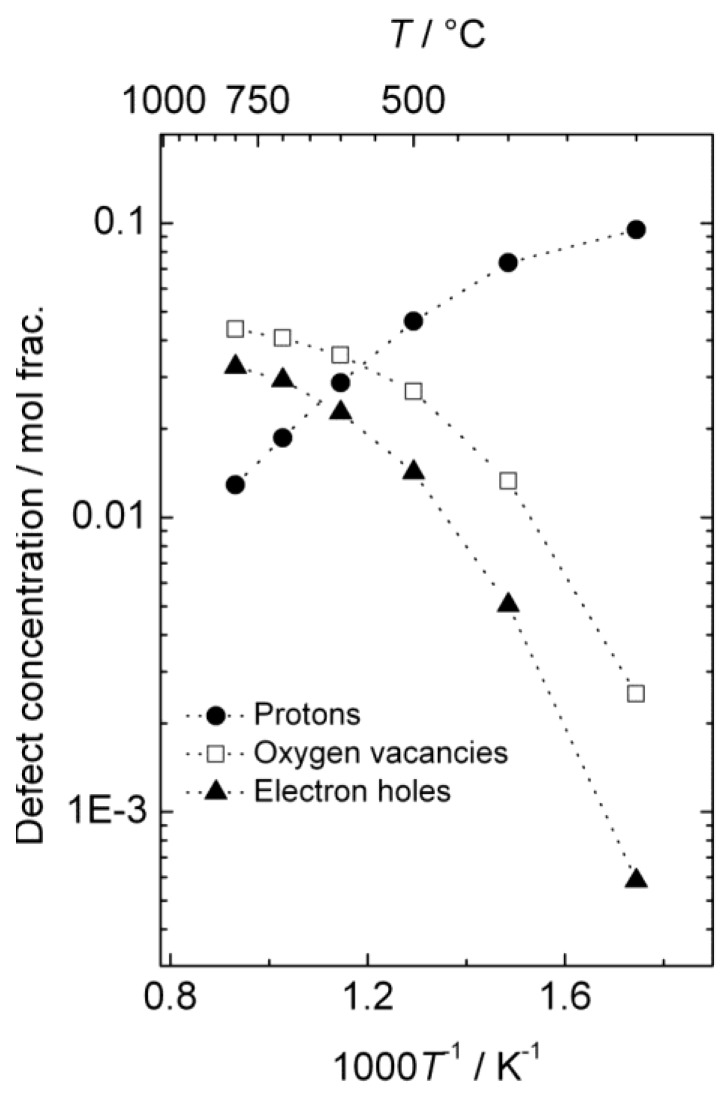
Estimated defect concentrations as a function of inverse temperature for La_0.9_Sr_0.1_CrO_3−*δ*_ in wet, reducing atmospheres (*p*H_2_ = 1 atm, *p*H_2_O = 0.025 atm).

**Figure 7:**
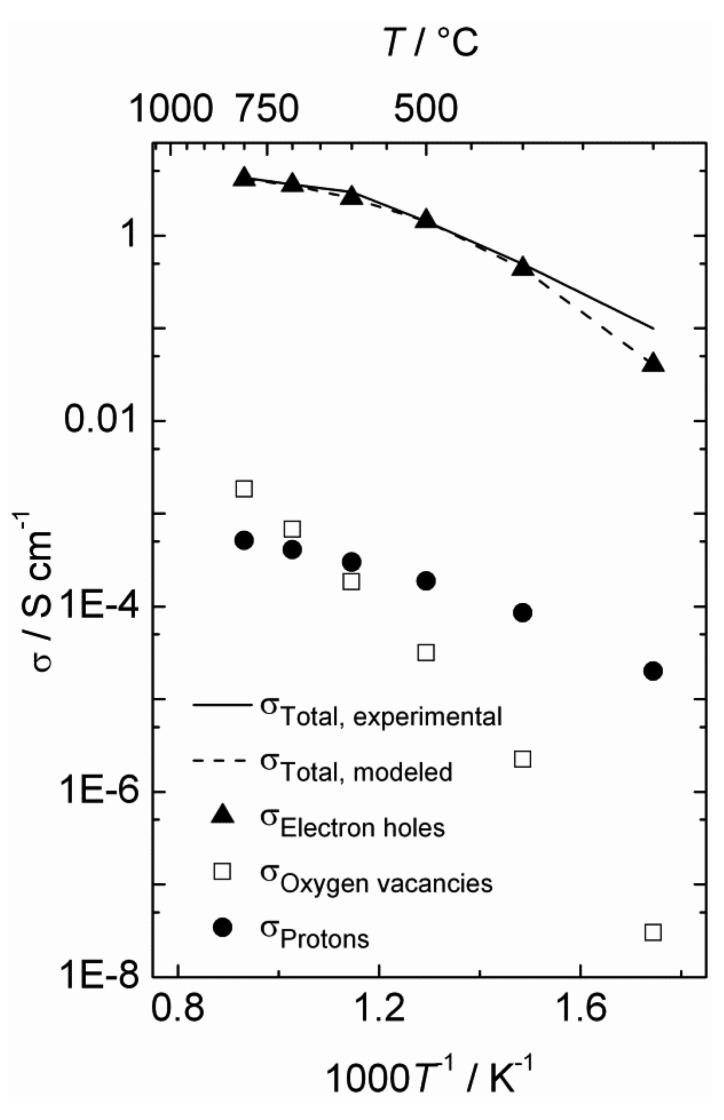
Estimated total and partial conductivities as a function of inverse temperature for La_0.9_Sr_0.1_CrO_3−*δ*_ in wet reducing atmospheres (*p*H_2_ = 1 atm, *p*H_2_O = 0.025 atm).

The estimated defect concentrations and partial conductivities are presented as a function of inverse temperature in Figure 6 and Figure 7, respectively. 

The estimated proton conductivity reaches approximately 5 × 10^−4^ S/cm at 800 °C, while the oxygen vacancies reach a conductivity in the order of 10^−3^ S/cm. Figure 7 also illustrates that the electron hole conductivity dominates the total conductivity, even when electron holes are minority defects under reducing conditions. The bends in the conductivity curves are the result of the changeover to a proton-dominated defect structure at lower temperatures.

Based on the estimated partial conductivities, we may estimate the ambipolar proton-hole conductivity of La_0.9_Sr_0.1_CrO_3−*δ*_ from

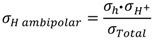
(6)
and it is limited by the lower of the two partial conductivities. In LSC, proton conductivity is orders of magnitude lower than that of electron holes, thereby limiting this ambipolar conductivity.

### 3.3. Measurements of Hydrogen Production with Disk-Shaped Membranes

**Figure 8:**
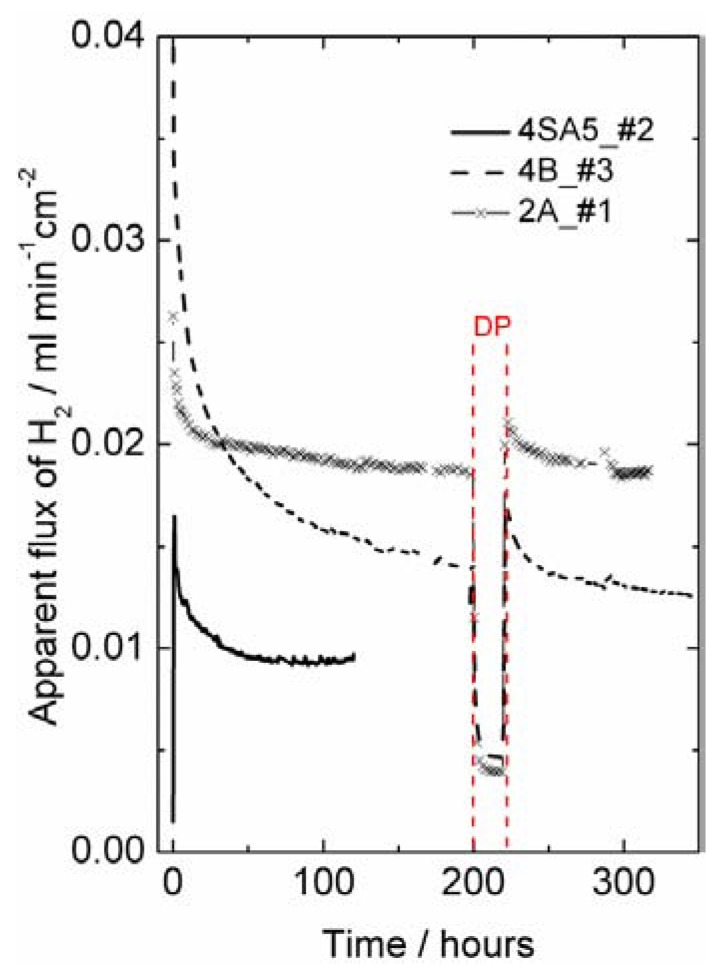
Hydrogen production plotted as apparent hydrogen (H_2_) flux (leakage corrected) as a function of time at 1000 °C using 50% wet H_2_ as feed gas for different LSC samples of thickness around 0.5 mm (see Table 1). Wet permeate gas is used for most of the experiment, except for a short period with dry permeate (DP), with a steep decrease in the hydrogen production rate.

Hydrogen production measurements—interpreted as apparent hydrogen flux—were performed on disk-shaped LSC membranes of approximately 0.5 mm thickness, prepared with various sintering agents (see Table 1). They showed an initial apparent flux of 0.04–0.02 mL min^−1^cm^−2^, which decreased over some 50–200 h at 1000 °C to final values of 0.02–0.01 mL min^−1^cm^−2^ at 1000 °C in 50% humidified H_2_ on the feed side and humidified argon at the permeate side (see Figure 8). The initially high apparent flux may be related to an initially high concentration of intrinsic defects created under the very high sintering temperature. The decrease in apparent flux may alternatively be related to the relaxation and decrease in surface catalytic activity of the polished surfaces during initial operation at elevated temperatures. 

**Figure 9:**
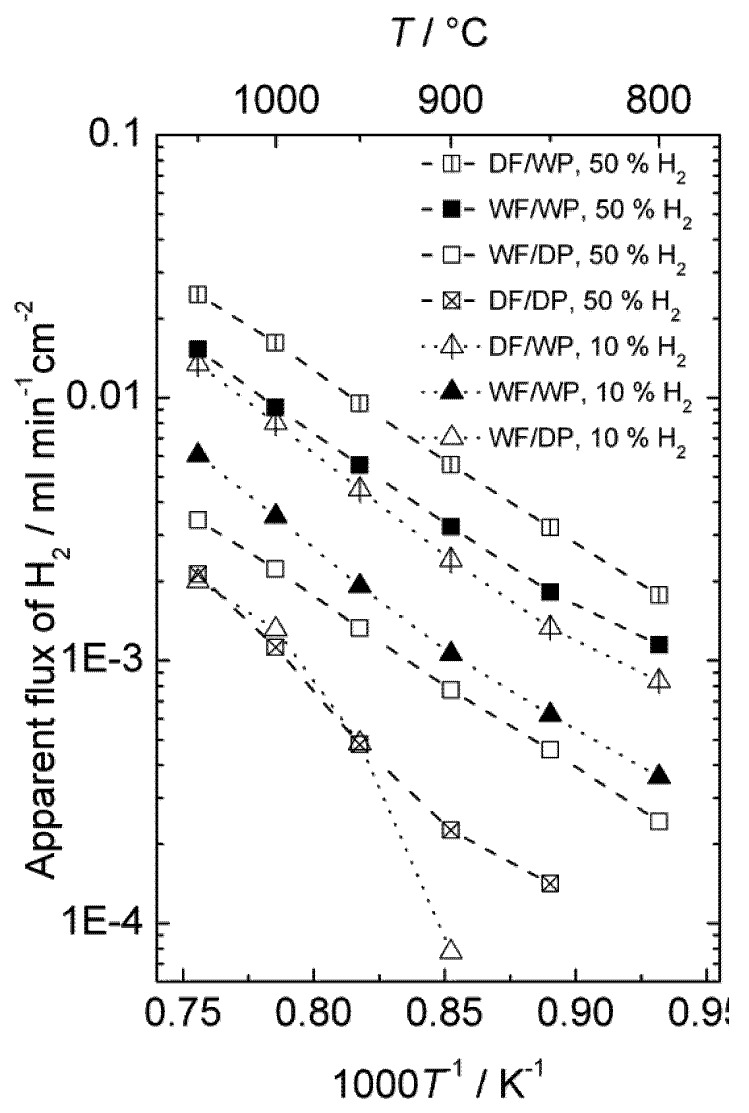
Hydrogen production plotted in terms of an apparent hydrogen (H_2_) flux as a function of inverse temperature for sample 4SA5_#2 in 4 different gradients using dry (D) or Wet (W) Feed (F) and Permeate (P) gas.

The effect of different types of gradients was further investigated in order to separate situations with only hydrogen (proton-hole) transport and cases where oxygen transport, and hence water splitting, contribute to the hydrogen production. Four different gradients are shown in Figure 9, where all have 50% or 10% hydrogen on the feed side and argon on the permeate side. The only difference is that the gases are either wet or dry. Accordingly, the following effects can be seen:

Dry feed/Wet permeate (DF/WP) gives the highest hydrogen production. This can be attributed to a high level of water splitting on the permeate side, combined with a high driving force for transport of oxygen from permeate to feed. Wet feed/wet permeate (WF/WP) gives a slightly lower hydrogen production. One can expect the driving force for water splitting to be lower, since the difference in *p*(O_2_) is less. Wet feed/dry permeate (WF/DP) gives yet lower hydrogen production. In this case we have no possibility of water splitting and the hydrogen must come from ambipolar proton transport. Dry feed/dry permeate (DF/DP) gives the lowest hydrogen production when comparing cases with equally amounts of hydrogen on the feed side. Here, we are probably additionally limited by the hydration and concentration of protons in the oxide.

It is noteworthy that what we believe is water splitting from ambipolar oxygen transport and what we believe is ambipolar hydrogen transport both appear to have similar and rather high activation energies. As we shall see later, this is possibly related to both being limited by surface kinetics of the polished disk samples used.

### 3.4. Measurements of Hydrogen Production with Disk-Shaped Membranes under Pressurized Conditions

**Figure 10:**
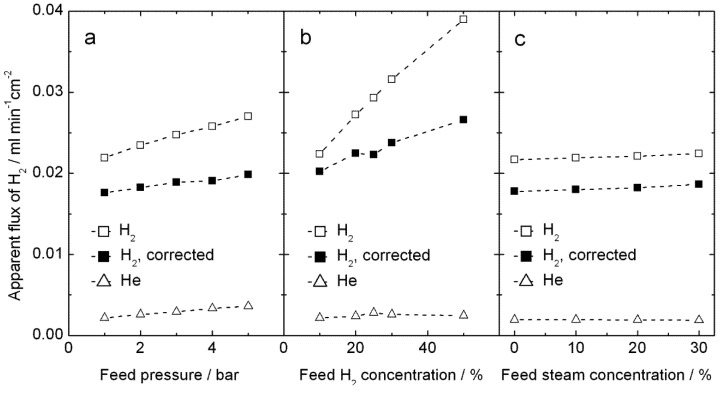
Measured apparent H_2_ flux, and the same corrected for leakage using the measured He flux, for sample 4B_#3 at 1000 °C, plotted as a function of (**a**) total feed pressure using H_2_/He/N_2_/steam = 20:10:50:20 on the feed side and Ar/steam = 80:20 on the permeate side,(**b**) feed hydrogen content *x* with H_2_/He/N_2_/steam = *x*:10:70 − *x*:20 on the feed side at 5 bar and Ar/steam = 80:20 on the permeate side at 4.9 bar, and (**c**) feed steam content *y* with H_2_/He/N_2_/steam = 20:10:50 − *y*:*y* on the feed side at 5 bar and Ar/steam = 80:20 on the permeate side at 4.9 bars.

Hydrogen production measurements were performed up to 5 bars on both feed and permeate sides, using high steam contents on both sides, in order to increase the proton concentration and reduce the vacancy concentration in LSC by water incorporation, according to Equation (3). Results for 1000 °C are plotted on Figure 10. The apparent hydrogen flux is largely independent of total pressure and of steam content, see Figure 10a, c, and instead the amount of hydrogen in the feed plays a somewhat larger role—see Figure 10b. These observations do not immediately lend support to either proton or oxygen transport; the hydrogen production can well come from both hydrogen flux and water splitting. We note, however, that the values of apparent flux are qualitatively similar to those done at atmospheric pressure and normal wet gases above. 

### 3.5. Measurements of Hydrogen Production with Monolith Module under Pressurized Conditions

**Figure 11:**
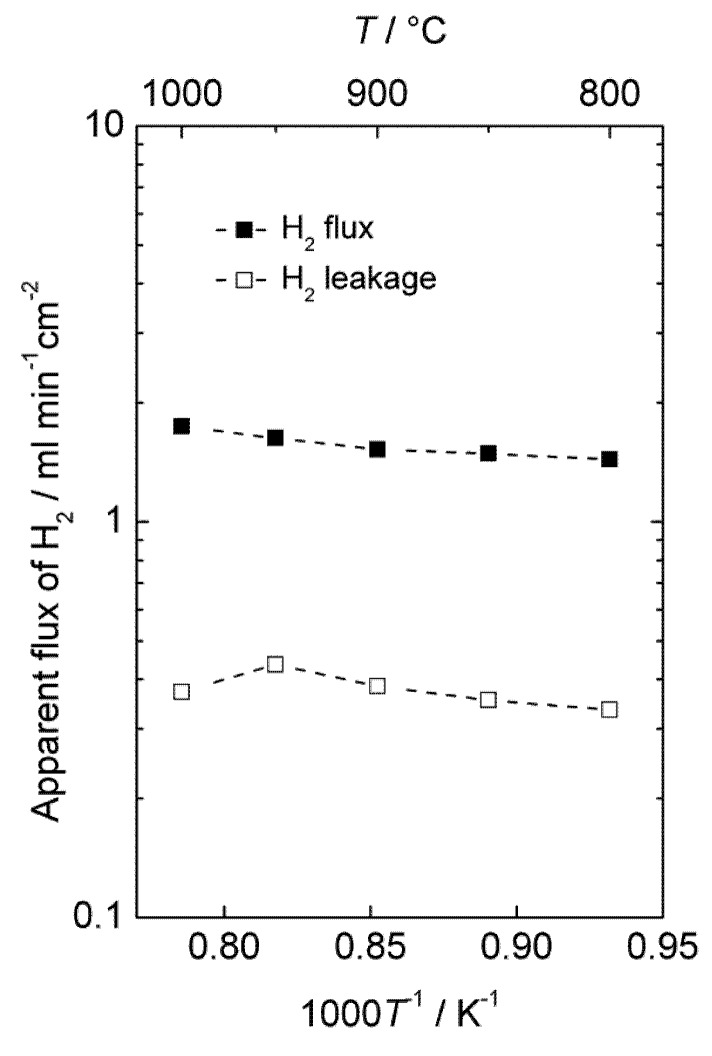
Apparent hydrogen flux and leakage of the monolith module at 20 bars pressure.

The hydrogen production of an LSC monolith module was also tested at 1000–800 °C and 20 bars pressure in moist hydrogen. The monolith had 37 active channels: 21 for feed gas and 16 for permeate sweep gas. The dense membrane was applied on the porous walls in the monolith on the sweep channel side and had a thickness of 50 μm. The concentration of hydrogen in the permeate gas was 19%–22%. Figure 11 shows the area specific hydrogen leakage and apparent hydrogen flux. 

Accordingly, there is a considerable apparent flux through the membrane, and the leakage is low compared to the calculated apparent hydrogen flux. The measurements were performed with co-current flow of feed and sweep gas, rapidly decreasing the driving force for hydrogen permeation, and the gas feeding rate (1000 mL/min) was also low compared to the high apparent flux and membrane area (86 cm^2^). Besides, the performance of the module was not tested with air on the sweep side, which would have served to maintain a high driving force throughout the monolith and increase the permeation of hydrogen, as mentioned in the introduction for the selected PCDC process. It is therefore expected that the module would perform even better under realistic conditions, e.g., counter-current flow regime with air at the sweep side. There is also room for decreasing the thickness of the membrane well below 50 µm. A hydrogen production rate of the order of 10 mL cm^−2^ min^−1^ under real operating conditions seems therefore to be within reach with this material. 

It may still be added that this experiment may also have had a large portion of the hydrogen produced coming from water splitting. The leakages were large enough to prevent a detailed analysis of the mass balances that could have answered this question.

We note that the apparent flux densities are now some two orders of magnitude larger than in the case of the polished disk samples and that the temperature dependency, *i.e.*, activation energy, is now small. We speculate that the surface of the membrane applied inside the monolith channels is catalytically active compared to the polished disk surfaces, and that the results of the monolith reflect bulk limited diffusion, while those of the disk samples reflect surface kinetics limitations. 

### 3.6. Flux Measurements Using D_2_ and D_2_O

**Figure 12:**
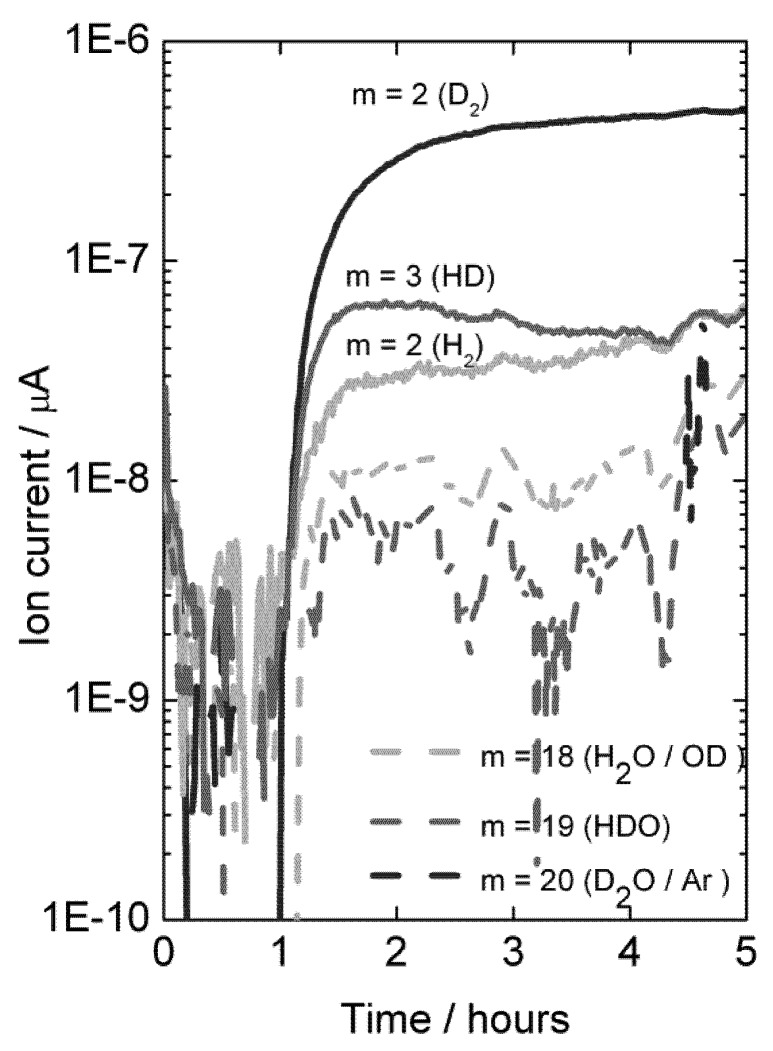
Mass spectrometer signal from various masses *vs.* time, indicative of deuterium flux through LSC disk membrane at 1040 °C when changing the feed gas from He + 2.5% D_2_O to D_2_ + 2.5% D_2_O after approximately 1 hour.

In order to unambiguously prove diffusion of hydrogen and perhaps quantify the possible contribution from water splitting on the permeate side of the membrane to the produced hydrogen, permeation measurements were carried out using D_2_ and D_2_O on the feed side of the membranes. Mass spectrometry was used to analyze the permeate gas exiting the cell. Results are plotted in Figure 12.

With dry permeate gas, the presence of only D_2_O on the feed side gives rise to very little D-containing species in the permeate, showing that there is little D_2_O flux from ambipolar proton (here deuteron) and oxide ion transport, see Figure 12 (first hour). Introduction of D_2_ on the feed side gives a driving force for ambipolar deuteron-electron hole transport, and a large and dominating increase in the D_2_ content on the permeate side results. This proves unambiguously the presence of hydrogen flux due to ambipolar proton (here deuteron) and electron hole transport.

**Figure 13:**
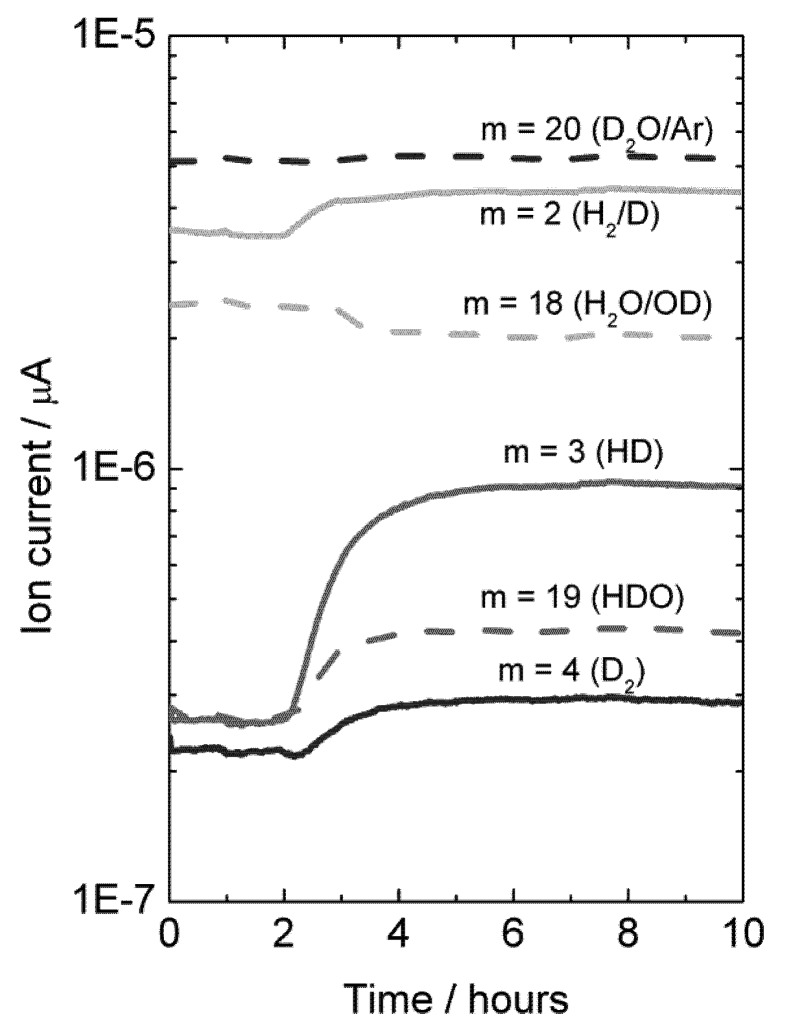
Mass spectrometer signal from various masses *vs.* time, indicative of deuterium flux through LSC disk membrane at 1040 °C when changing the feed gas from He + 2.5% D_2_O to D_2_ + 2.5% D_2_O after approximately 2 hours. Permeate gas is Ar + 2.5% H_2_O.

Experiments were also made with H_2_O-wetted permeate gas. In this case, D_2_ in the permeate, originated from flux, will isotopically mix with H_2_O, resulting in an increase in the concentration of species HD, HDO, H_2_ and D_2_O, and a decrease in the concentration of H_2_O, see Figure 13. 

A mass balance analysis of the permeate flue gas for this experiment at 1040 °C results in around 80% of the hydrogen (here hydrogen and deuterium) produced comes from hydrogen (here deuterium) flux and that 20% comes from water splitting. 

It may be noted that the sample used here was again a polished disk membrane. The high level of hydrogen flux compared to water splitting suggests that although both processes may be surface kinetics limited, the water splitting is more limited by surface kinetics than the hydrogen flux is. 

### 3.7. Comparison of Data from Different Techniques

**Figure 14:**
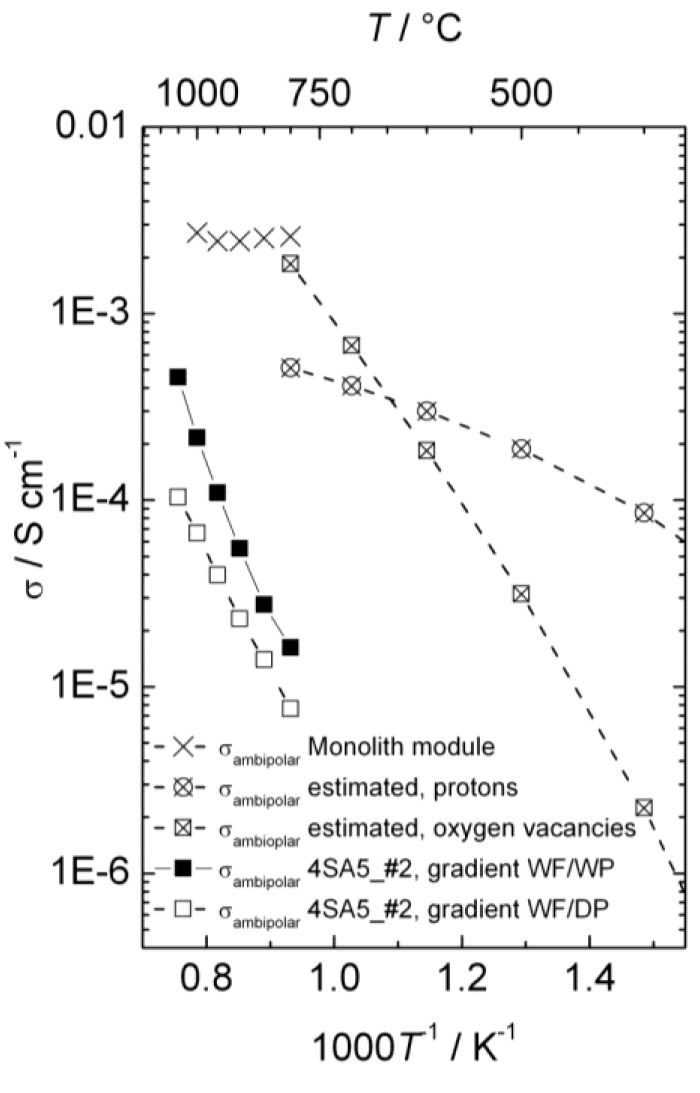
Ambipolar proton-electron hole and oxygen vacancy-electron hole conductivity as a function of inverse temperature for La_0.9_Sr_0.1_CrO_3−*δ*_, estimated from conductivity measurements, compared with conductivities extracted from measurements of hydrogen production with disk membrane in two different gradients and with the monolith module. All proton conductivities are extrapolated to 0.025 atm H_2_O.

Figure 14 presents the ambipolar proton-electron hole and oxygen vacancy-electron hole conductivities as a function of inverse temperature estimated from electrical measurements, the ambipolar proton-electron hole conductivity extracted from the measurements of hydrogen production with the disk membrane (wet and dry permeate gas), and the ambipolar proton-electron hole conductivity extracted from the measurements of hydrogen production with the monolith module (wet permeate gas). 

Two different models were used to describe the hydrogen flux and relate it to ambipolar conductivity for wet and dry permeate gas. Both assume that the transport number for electron holes is unity, so that ambipolar conductivity equals proton conductivity. For wet permeate gas (and wet feed gas) we assume that the hydration level and thus proton concentration is constant throughout the membrane. The difference in *p*H_2_ is the only driving force for hydrogen (proton) flux, and the following model is applied:

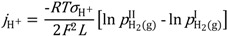
(7)

Here, the proton conductivity is the one valid for the *p*H_2_O used in the measurement, *L* is the membrane thickness, and the other symbols have their usual meanings.

From the dry permeate case, we assume that the *p*H_2_/*p*H_2_O ratio is constant throughout the membrane, which is attained to a first approximation through ambipolar water flux accompanying the hydrogen flux. The proton conductivity is thus not constant throughout the membrane, and the following model is applied:

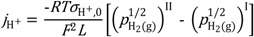
(8)

Here 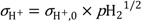 the *p*H_2_/*p*H_2_O used. It may be noted that both models yield proton conductivities that are valid for a given water vapor content, which may be recalculated for another one assuming that the protons are minority defects and their concentration proportional to *p*H_2_O^1/2^, and this has been applied in plotting Figure 14. 

While the models are uncertain, the choice of model does not dramatically alter the resulting proton conductivity.

Based on the knowledge of the defect structure and transport properties of LSC, the conductivity measurements, the D_2_ flux measurements and isotope exchange data, and the hydrogen production measurements from disk and monolith membranes, we may now tentatively conclude the following from our studies so far:

From the changes in p-type electronic conductivity with *p*H_2_O, we know that LSC hydrates significantly and contains considerable concentrations of protons. The thermodynamics of hydration can be extracted, and we can make a first guess of the proton conductivity in LSC in wet atmospheres as a function of temperature and compare this with an estimate of the oxide ion conductivity which is based on more solid data (Figure 14). From this and the dominating p-type electronic conductivity we can predict that LSC will be permeable to hydrogen, oxygen, and water in gradients of *p*H_2_, *p*O_2_, and *p*H_2_O. The two first can be used respectively for hydrogen production by hydrogen flux and water splitting. 

The D_2_ flux measurements prove unambiguously that there is in fact hydrogen flux in the material. Moreover, they suggest that hydrogen flux and water splitting contribute within the same order of magnitude to the hydrogen production (80% and 20%, respectively, for a disk sample at 1040 °C in wet gases). 

Hydrogen production measurements using a monolithic membrane module with large area at high pressures and high steam contents yield a high output, one order of magnitude higher than estimated from the guessed proton conductivity, and with a small temperature dependency as predicted for hydrogen flux limited by bulk transport, see Figure 14. All in all, we suggest that this is to a large extent hydrogen flux, and that the protons therefore may have a higher mobility than guessed.

Hydrogen production measurements using polished disk samples show variations with atmosphere composition that support the simultaneous presence of hydrogen flux and water splitting. The activation energies are, however, consistently higher and the apparent flux lower than expected from the ambipolar conductivities (see Figure 14). This suggests that these samples are severely limited by surface kinetics. Based on this, the activation energy for surface kinetics is similar for both hydrogen flux and water splitting, although details in the results from the various experiments can be interpreted to reflect that water splitting is more limited by surface kinetics than hydrogen permeation.

The monolith module demonstrated promising flux densities and hydrogen production rates, and appears to have been produced with relatively active surfaces.

Future studies of LSC membranes for hydrogen production must focus on the kinetics and engineering of its surfaces. 

## 4. Experimental Section

### 4.1. Samples Preparation

The LSC powders were prepared by spray pyrolysis at 750 °C of an aqueous nitrate solution with chromium, strontium and lanthanum concentrations according to the perovskite stoichiometry. The solution contained 1 mole metal ions per liter, corresponding to approximately 115 g perovskite per liter. To retain maximum sintering activity and suppress the concentration and evaporation of carcinogenic Cr(VI), the powder was calcined in vacuum (1 mbar) at 850 °C. The powder was finally milled in a steel attrition mill for 120 min at 300 rpm with 3 kg distilled water to 2 kg LSC. To increase sinterability, small additions (2–4 mol%) of various partly-proprietary sintering aids were added during milling. Dilatometer studies of a powder batch with 2 mol% CaMnO_3_ demonstrated densities of >95% by sintering in air at 1700 °C or in 4% H_2_ in N_2_ at 1600 °C. The latter condition was used for sample preparation, where the LSC powder with various sintering aids (Table 1) was pressed into green bodies and sintered to dense pellets.

**Table 1:** Overview of samples.

Short name	Composition	Sintering aid mol%	Thickness [mm]	Diameter [mm]
2A_#1	La_0.87_Sr_0.13_CrO_3_	2 ("A")	0.435	20
4B_#3	La_0.87_Sr_0.13_CrO_3_	4 ("B")	0.445	20
4SA5_#2	La_0.87_Sr_0.13_CrO_3_	4 (CaMnO_3_)	0.550	20

LSC powder with 2 mol% CaMnO_3_ was also used for fabrication of the small asymmetric monolithic membrane module. The support structure was a circular porous monolith, extruded from a mixture of LSC and pore former (20 wt % cellulose fiber) with binder (methyl cellulose), plasticizer (polyethylene glycol) and water. The extruded monoliths were dried at 40 °C, while a fan maintained air circulation through the channels. To remove the organic components, the dry monoliths were heated to 500 °C at a low rate of 0.1°/min to avoid overheating and cracking. After a final pre-sintering of the support monolith at 1200 °C, the LSC membrane was applied by dip coating with access to each second monolith channel in a chess pattern. After sintering in dilute hydrogen at 1700 °C, the dense membrane layer was 50 µm thick, and proved gas impervious at room temperature, while the channel width and wall thickness were 1.7 and 0.4 mm, respectively. 

Pictures of the membranes and the monolith have been published elsewhere [3]. The monolith had 37 active channels: 21 for feed gas and 16 for sweep gas. The membrane was applied in the sweep channels. The module was made from a 90 mm long monolith with 86 cm^2^ membrane area. To distribute feed and sweep gas in the appropriate channels, manifolds were sealed to the monolith at each end. Each manifold was made from a dense choke plate and gas distributor in LSC and a hat in yttrium stabilized zirconia. The components were sealed to each other by a glass ceramic with thermal expansion profile matching LSC. The module also had 2 gas baffles in platinum around the monolith, matching the inner diameter of the test reactor. During testing, the hats were sealed to Al_2_O_3_ support tubes by gold rings. Thus, the feed side of the membrane module was available from the inside of the supply tubes, while the sweep channels were available from the outside through the side holes of the gas distributor. 

### 4.2. Electrical Conductivity and Measurements of Hydrogen Production

Electrical conductivity measurements in controlled atmospheres were performed on a La_0.9_Sr_0.1_CrO_3_-sample in a ProboStat^TM^ cell (NorECs AS, Norway). The sample was rather porous—only 82% dense—in order to get a fast response with changes of atmosphere. An alternating current (a.c.), van der Pauw four point method, was used to measure the conductivity as a function of temperature and concentration of water vapor. The measurements were performed using a Solartron SI 1260 Frequency Response Analyzer operating at 1590 Hz and 1 V amplitude. The conductivity was not corrected for porosity, as this is a minor effect, and has no consequence for the interpretations used here. Different hydrogen-argon mixtures were used and the gas composition was controlled by gas mixing and wetting stages. The conductivity measurements were done under atmospheric pressure.

Measurements of hydrogen production of disk-shaped membranes were performed in a ProboStat^TM^ cell, using planar polished samples and gold rings as seals. The support tubes were also polished, in order to improve the sealing process. Helium was used to verify successful sealing, indicated by <0.01 mol% detection of He using a gas chromatograph. Measurements were performed by using a humidified He:H_2_ mixture (50:50 and 90:10) on the feed side (with a total of 50 mL/min gas flow) and humidified Ar on the permeate side (with a gas flow of 25 mL/min). For pressurized measurements to 5 bars, the normal flow was multiplied by the pressure. Both He and H_2_ concentrations were measured with a calibrated Varian 4900 µ-GC. The apparent fluxes were calculated from the measured permeate H_2_ concentration and the calibrated flow of Ar sweep gas after correcting for He leak using a Knudsen ratio. For the pressurized flux measurements, a metal mantel was used on the ProboStat^TM^ cell, which allowed measurement up to 30 bars at 900 °C or 10 bars at 1000 °C. For mass spectrometry measurements, the permeate gas was analyzed with a Pfeiffer PrismaPlus QMG 220, and the spectrometer ion current converted to relative concentrations of species in the gas, based on several background and calibration runs of known gas mixtures. 

Measurements of hydrogen production of the module were carried out in a different rig. The module was made from a 9 cm-long monolith, which gave a total of 86 cm^2^ membrane area. It was installed in a reactor of Paralloy CR39W steel alloy, which is suited for test conditions involving high pressure, temperature and hydrogen activity. The feed gas was a mixture of 70% H_2_, 20% steam, and 10% N_2_, while the sweep gas was 80% Ar and 20% steam. At each side, the total flow rate was 1000 mL/min. After sealing the monolith module to the test reactor at 1000 °C, only 0.5% nitrogen was detected in the sweep side flue gas in the entire temperature range.

## 5. Conclusions

In this study, we have evaluated the theoretical and empirical background for the potential use of acceptor-doped LaCrO_3_ as a hydrogen permeable membrane for hydrogen production, based on mixed proton and electron hole conductivity. We have also taken into account the possibility of oxygen permeation producing hydrogen from water splitting as a competing mechanism. We have reported on experimental studies of hydrogen production rates of LSC membranes prepared as dense polished discs and advanced monolithic geometry in various atmospheres, including pressurized conditions. Both gas chromatography and mass spectrometry using deuterium were carried out. The results confirm unambiguously that there is hydrogen flux by ambipolar proton and electron hole transport and show that oxygen transport and water splitting contribute. For the monolith module, the hydrogen fluxes are higher than predicted from estimates of proton conductivity, and have potential for further improvement (thinner membrane and better flow patterns), making LSC-based membranes promising for hydrogen production. Our studies indicate that surface kinetics easily become limiting for LSC for hydrogen permeation and probably even more so for water splitting, and studies of this and engineering of surfaces stand out as crucial in future developments. 

## References

[1] Middleton P., Hurst P., Walker G., Thomas D.C., Benson S. (2005). GRACE: Pre-Combustion De-Carbonisation Hydrogen Membrane Study. Carbon Dioxide Capture for Storage in Deep Geologic Formations: Results from the CO_2_ Capture Project.

[2] Beavis R. (2011). The EU FP6 CACHET project—Final results. Energy Procedia.

[3] Smith J.B., Aasen K.I., Wilhelmsen K., Käka D., Risdal T., Berglund A., Stenersen Østby A., Budd M., Bruun T., Werswick B. (2009). Recent development in the HMR pre-combustion gas power cycle. Energy Procedia.

[4] Fontaine M.L., Norby T., Larring Y., Grande T., Bredesen R. (2008). Oxygen and hydrogen separation membranes based on dense ceramic conductors. Membr. Sci. Technol..

[5] Norby T., Haugsrud R., Sammells A.F., Mundschau M.V. (2006). Dense Ceramic Membranes for Hydrogen Separation. Nonporous Inorganic Membranes.

[6] Davies R., Islam M., Gale J. (1999). Dopant and proton incorporation in perovskite-type zirconates. Solid State Ionics.

[7] Norby T., Widerøe M., Glöckner R., Larring Y. (2004). Hydrogen in oxides. Dalton Trans..

[8] Norby T., Ishihara T. (2009). Proton Conductivity in Perovskite Oxides. Perovskite Oxide for Solid Oxide Fuel Cells.

[9] Bjørheim T.S., Kuwabara A., Ahmed I., Haugsrud R., Stølen S., Norby T. (2010). A combined conductivity and DFT study of protons in PbZrO_3_ and alkaline earth zirconate perovskites. Solid State Ionics.

[10] Glöckner R. (2000). Dissolution and Transport of Protons in Some Perovskite-Related Oxides. Ph.D Thesis.

[11] Iwahara H. (1992). Oxide-ionic and protonic conductors based on perovskite-type oxides and their possible applications. Solid State Ionics.

[12] Solís C., Escolastico S., Haugsrud R., Serra J.M. (2011). La_5.5_WO_12−δ_ characterization of transport properties under oxidizing conditions: A conductivity relaxation study. J. Phys. Chem. C.

[13] Julsrud S., Vigeland B.E. (2007). A Solid Multicomponent Mixed Proton and Electron Conducting Membrane. EP Patent.

[14] Aasen K., Vigeland B., Norby T., Larring Y., Mejdell T. (2005). Development of a hydrogen membrane reformer based CO_2_ emission free gas fired power plant. Greenhouse Gas Control Technol. 7.

[15] Tezuka K., Hinatsu Y., Nakamura A., Inami T., Shimojo Y., Morii Y. (1998). Magnetic and neutron diffraction study on perovskites La_1−*x*_Sr*_x_*CrO_3_. J. Solid State Chem..

[16] Schneider S., Roth R., Waring J. (1961). Solid state reactions involving oxides of trivalent cations. J. Res. Natl. Bur. Stand..

[17] Peck D., Miller M., Hilpert K. (1999). Phase diagram study in the CaO–Cr_2_O_3_–La_2_O_3_ system in air and under low oxygen pressure. Solid State Ionics.

[18] Peck D., Miller M., Hilpert K. (1999). Phase diagram studies in the SrO–Cr_2_O_3_–La_2_O_3_ system in air and under low oxygen pressure. Solid State Ionics.

[19] Zhu W., Deevi S. (2003). Development of interconnect materials for solid oxide fuel cells. Mater. Sci. Eng. A.

[20] Mizusaki J., Yamauchi S., Fueki K., Ishikawa A. (1984). Nonstoichiometry of the perovskite-type oxide La_1−*x*_Sr*_x_*CrO_3_-[delta]. Solid State Ionics.

[21] Flandermeyer B., Nasrallah M., Agarwal A.K., Anderson H. (1984). Defect structure of mg‐doped lacro_3_ model and thermogravimetric measurements. J. Amer. Ceram. Soc..

[22] Yasuda I H.T., Grosz F Z.P., Singahl S.C., Yamamoto O. (1991). Electrical Conductivity and Oxygen Chemical Diffusion Coefficients of Calcium-Doped Lanthanum Chromites, Second International Symposium on Solid Oxide Fuel Cells.

[23] Yasuda I., Hikita T. (1993). Electrical conductivity and defect structure of calcium‐doped lanthanum chromites. J. Electrochem. Soc..

[24] Fergus J.W. (2007). Materials challenges for solid-oxide fuel cells. JOM J. Min. Metal. Mater. Soc..

[25] Koc R., Anderson H.U. (1992). Liquid phase sintering of LaCrO_3_. J. Eur. Ceram. Soc..

[26] Hilpert K., Das D., Miller M., Peck D., Weiss R. (1996). Chromium vapor species over solid oxide fuel cell interconnect materials and their potential for degradation processes. J. Electrochem. Soc..

[27] Yokokawa H., Sakai N., Horita T., Yamaji K. (2001). Recent developments in solid oxide fuel cell materials. Fuel Cells.

[28] Paulik S., Baskaran S., Armstrong T. (1998). Mechanical properties of calcium-and strontium-substituted lanthanum chromite. J. Mater. Sci..

[29] Fergus J.W. (2005). Metallic interconnects for solid oxide fuel cells. Mater. Sci. Eng. A.

[30] Yasuda I., Hishinuma M. (1996). Electrochemical properties of doped lanthanum chromites as interconnectors for solid oxide fuel cells. J. Electrochem. Soc..

[31] Karim D., Aldred A. (1979). Localized level hopping transport in La(Sr)CrO_3_. Phys. Rev. B.

[32] Yokokawa H., Horita T., Sakai N., Hassel B. Oxygen Permeation and Related Phenomena of Lanthanum Calcium Chromites as SOFC Interconnects. Proceedings of the 3rd International Symposium on Solid Oxide Fuel Cells.

[33] Van Hassel B.A., Kawada T., Sakai N., Yokokawa H., Dokiya M. (1993). Oxygen permeation modelling of La_1−*y*_Ca*_y_*CrO_3−*∂*_. Solid State Ionics.

[34] Kawada T., Horita T., Sakai N., Yokokawa H., Dokiya M. (1995). Experimental determination of oxygen permeation flux through bulk and grain boundary of La_0.7_Ca_0.3_CrO_3_. Solid State Ionics.

[35] Weber W.J., Griffin C.W., Bates J.L. (1987). Effects of cation substitution on electrical and thermal transport properties of YCrO_3_ and LaCrO_3_. J. Amer. Ceram. Soc..

